# The Efficacy and Safety of Modified Docetaxel, Cisplatin, and 5-Fluorouracil Vs. Epirubicin, Oxaliplatin, and Capecitabine Regimen in the Advanced Gastric Cancer: A Randomized Controlled Clinical Trial

**DOI:** 10.31557/APJCP.2020.21.3.727

**Published:** 2020-03

**Authors:** Ahmad Ahmadzadeh, Seyed Saeid Seyedian, Armita Valizadeh, Mahin Soleimani, Pedram Nazari, Hossein Hamidi

**Affiliations:** 1 *Research Center of Thalassemia and Hemoglobinopathy, *; 2 *Alimentary Tract Research Center, *; 3 *Department of Anatomy, *; 4 *Student Research Committee, *; 5 *Cancer Research Center, Ahvaz Jundishapur University of Medical Sciences, Ahvaz, Iran. *

**Keywords:** Gastric cancer, EOX, m-DCF, efficacy, safety

## Abstract

**Introduction:**

One of the major challenges of advanced gastric cancer treatment is the lack of a standard regimen for patients. However, several clinical trials have shown that modified docetaxel, cisplatin, and 5-fluorouracil (m-DCF) and epirubicin, oxaliplatin, and capecitabine (EOX) regimens are superior to other regimens.

**Methods:**

This randomized, single-center clinical trial was performed on 40 patients with advanced gastric cancer. The first group received the m-DCF regimen as follows: docetaxel (40 mg/m^2^) on the first day; cisplatin (40 mg/m^2^) on the first and second days; and 5-fluorouracil (400 mg/m^2^) from the first to fourth day. The second group received the EOX regimen, including epirubicin (50 mg/m^2^) and oxaliplatin (130 mg/m^2^) i.v on the first day and capecitabine at a twice-daily dose of 625 mg/m^2^ p.o for 21 days. Treatment was applied every three weeks for a total of eight cycles in both groups. In each group, the overall and progression-free survival rates and toxicity were assessed.

**Results:**

A total of 40 patients were enrolled in this study (21 samples in the m-DCF group and 19 samples in the EOX group), 62.5% of whom were male. The median survival rate was 14.00 (95% CI: 11.82-16.18) months in the m-DCF group and 15.00 (95% CI: 9.56-20.43) months in the EOX group; however, differences between the groups were not significant. The progression-free survival rate was higher in the EOX group, although there was no significant difference between the two groups. Also, there was no significant difference regarding the side effects (e.g., toxicity) or need for supportive care between the groups.

**Conclusion:**

It seems that both m-DCF and EOX regimens are similar in terms of survival and toxicity and are recommended as first-line treatment for advanced gastric cancer with respect to the patient’s status.

## Introduction

Despite all advances in the prevention, diagnosis, and treatment of gastric cancer, one million new cases were diagnosed in 2018, and 783,000 people died because of the disease. Considering the serious consequences of gastric cancer, it can alone undermine all advances made in this area (Bray et al., 2018). Since the disease continues to be asymptomatic until advanced stages, the main treatment options are practically limited to palliative three-drug chemotherapy regimens (Nishiyama and Wada, 2009; Van et al., 2011; Kuo et al., 2014).

In first-line palliative three-drug chemotherapy, different regimens, with various drugs and doses, have been suggested. However, there is still a gap in knowledge, and none of the proposed agents have been approved as the gold standard of treatment for advanced gastric cancer; therefore, there is an urgent need to improve and correct these regimens (de Gramont et al., 1988; de Gramont et al., 1997; Özdemir et al., 2010; Shah et al., 2010; Unek et al., 2013; Kalinka-Warzocha et al., 2015). The modified docetaxel, cisplatin, and 5-fluorouracil (m-DCF) regimen is one of the most common regimens, which uses adjusted doses of docetaxel, cisplatin, and 5-fluorouracil, with the aim of reducing hematological and gastrointestinal toxicity caused by factor D (5-10). The efficacy of m-DCF regimen has been approved in several clinical trials for advanced gastric cancer (de Gramont et al., 1988; de Gramont et al., 1997; Shah et al., 2010; Unek et al., 2013; Kalinka-Warzocha et al., 2015). Many researchers have used this regimen, alone or in combination with other chemotherapy agents, to treat advanced gastric cancer and confirmed its safety and efficacy (Unek et al., 2013). Despite the acceptable level of toxicity associated with this regimen, there are still uncertainties about its application, which indicate the need for future studies to evaluate different doses of DCF (de Gramont et al., 1997; Cunningham et al., 2008; Shah et al., 2010; Unek et al., 2013; Kalinka-Warzocha et al., 2015; Ochenduszko et al., 2015)

Another regimen used in some oncologic centers as first-line treatment for gastric and gastroesophageal junction cancers is epirubicin, oxaliplatin, and capecitabine (EOX) regimen, which showed a significantly higher efficacy in REAL2 clinical trial, compared with the ECF regimen. In this trial, the survival rate was 11.2 months in EOX patients versus 9.9 months in ECF patients. This regimen is better tolerated by patients than ECF and is associated with fewer thromboembolic events; also, it does not need a central catheter insertion, unlike DCF. Nevertheless, its side effects may cause problems for patients with cardiac and renal diseases or those with dysphagia. In addition, the role of epirubicin remains controversial (Ochenduszko et al., 2015). 

Although different studies have been conducted on the efficacy, safety, and tolerability of m-DCF and EOX regimens, few studies have compared these two regimens. Therefore, we designed and performed this trial to determine the optimal chemotherapy regimen for patients with stage IV gastric cancer.

## Materials and Methods

This study was a randomized clinical trial conducted between 2016 and 2019 in the Oncology Department of Shafa Hospital, as the largest tertiary referral center of oncology in southwest of Iran. The participants were patients with advanced (inoperable) or metastatic gastric adenocarcinoma, whose diseases were confirmed by histological studies.

*Eligibility criteria*

Advanced gastric cancer patients, aged 18 to 65 years with an Eastern Cooperative Oncology Group (ECOG) status ≤ 1, adequate cardiac, pulmonary, and renal function, and lack of specific psychological, familial, social, and geographical situations that may affect compliance and adherence to treatment, were included. Patients with sensory peripheral neuropathy ≥ grade 1 (according to CTCAE version 3.0), myocardial infarction, unstable angina for less than six months before the start of the study, stroke or pulmonary embolism, uncontrolled infection, pregnancy or lactation, and history of other malignancies and chemotherapy were excluded.

*Interventions*

Before the beginning of the study, the trial was first approved by the ethics committee of Ahvaz Jundishapur University of Medical Sciences with the Ethics Committee reference number, Ir.ajums.rec.1395.763. The trial was also registered in the Iranian Registry of Clinical Trials (IRCT2017062134670N1) at 07/08/2017. In addition, voluntary informed consent was obtained from all patients according to the Declaration of Helsinki.

Eligible subjects were randomly divided into two groups by block randomization method, and finally, 21 patients in the m-DCF arm and 19 patients in the EOX arm were enrolled. The investigator responsible for randomization was not involved in the assessment of efficacy and safety of regimens. Assessments before the start of treatment included history-taking, physical examination, complete blood cell count (CBC), coagulation tests (PT and PTT), and serum biochemistry and liver function tests (LFT). One month before the intervention, chest and abdominopelvic CT scans (with or without upper gastrointestinal tract endoscopy) were performed.

The first group received the m-DCF regimen as follows: docetaxel (40 mg/m^2^; Sanofi, France), diluted in 500 mL of normal saline and infused intravenously over 60 minutes on the first day; cisplatin (40 mg/m^2^; Mylan, France), diluted in 1,000 mL of normal saline on the first and second days and infused intravenously over 60 minutes; and 5-fluorouracil (400 mg/m^2^; Ebewe, Australia), administered via central venous catheters from the first to fourth day. 

For the second group, the EOX regimen was administered as follows: epirubicin (50 mg/m^2^; Ebew, Austria) as intravenous bolus on the first day; oxaliplatin (130 mg/m^2^; Sanofi, France), infused over two hours on the first day; and capecitabine (625 mg/m^2^; Roche, Switzerland) on 21 days p.o. For both regimens, common treatments to prevent nausea and vomiting were applied.

The treatment cycle for both m-DCF and EOX treatment regimens was repeated every three weeks for a maximum of eight cycles, except for samples with disease progression, unacceptable toxicity, withdrawal from the study, or death. Researchers graded all side effects and toxic effects in patients according to the criteria of the National Cancer Institute (version 2.0) in each visit.

*Toxicity assessment*

After completing the treatment, the patients were followed-up within three-month intervals for two years. Disease progression was assessed using physical examinations, carbohydrate antigen 19-9 (CA 19-9) and carcinoembryonic antigen tests, chest radiography, abdominal ultrasonography, and, if necessary, abdominopelvic CT scan or MRI.

*Statistical analysis*

Demographic data and other continuous variables are reported as mean±standard deviation, median, and range. Progression-free survival (PFS) was defined as the length of time from entering the study until the first report of relapse or the latest follow-up. Also, overall survival (OS) was defined as the length of time from the onset of chemotherapy to death or the latest follow-up. OS and PFS were calculated by Kaplan-Meier method and compared between the two groups, based on log-rank test. P-value less than 0.05 was considered significant. Data analysis was performed in SPSS version 14.

## Results

**Figure 1:**
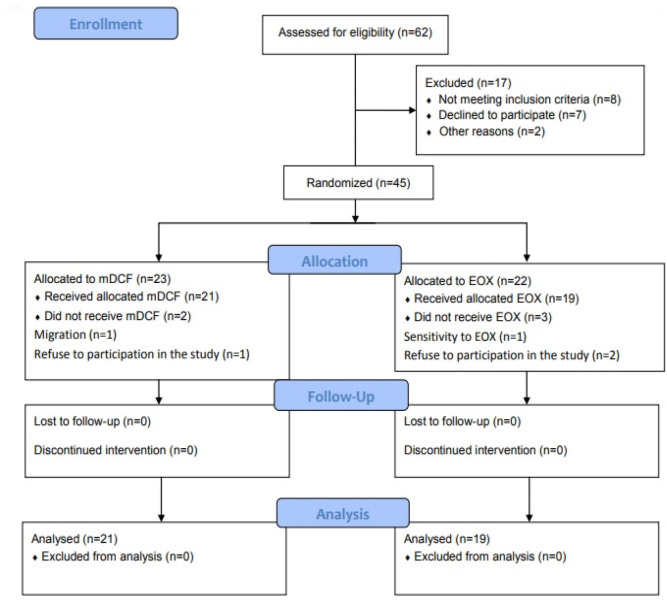
CONSORT Flow Diagram of Trial

In this study, 62 patients with gastric cancer were assessed for eligibility criteria. Forty-five patients met the inclusion criteria and were divided into the m-DCF (n=21) and EOX (n=19) groups (Figure 1). Twenty-five (62.5%) patients were male, while the rest were female. The mean age of the participants was 58.26±15.39 years. Both treatment groups were almost homogeneous in terms of the baseline characteristics, except for the mean age (P= 0.08) and liver metastasis (P= 0.06); however, the differences were not significant. The baseline characteristics of the patients are presented in Table 1.

*Patient survival (primary endpoint)*

**Figure 2:**
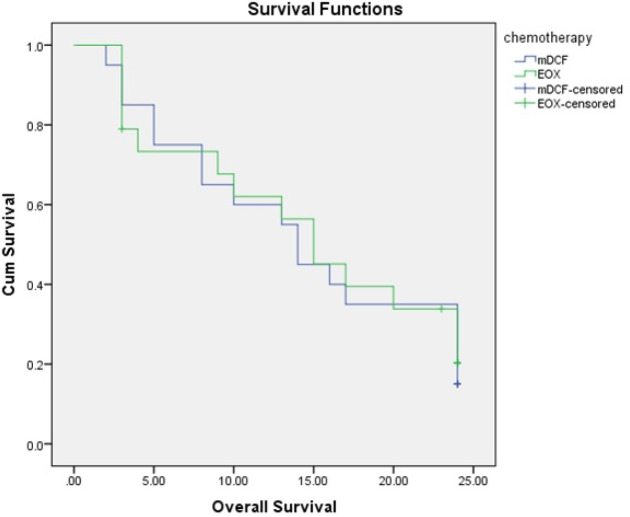
Kaplan–Meier Estimates of Ooverall Survival

**Figure 3:**
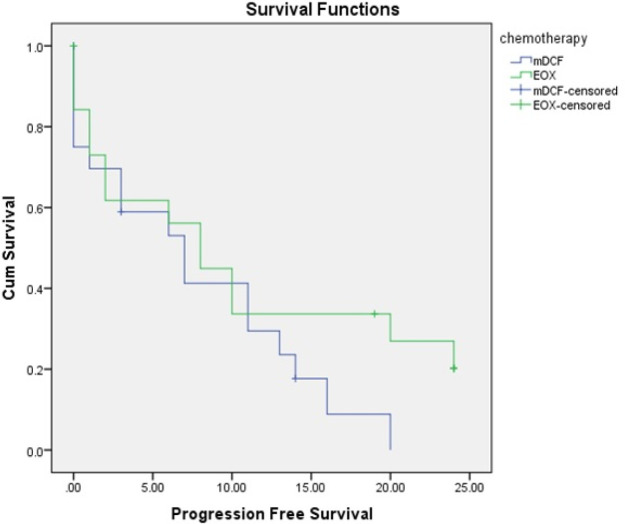
Kaplan–Meier Estimates of Progression-free Survival

As shown in Table 2, the median survival in the m-DCF and EOX groups was 14.00 (95% CI: 11.82-16.18) and 15.00 (95% CI: 9.56-20.43) months, respectively; however, the difference was not statistically significant between the groups. Also, the two-year survival rate in the EOX group was higher than that of the m-DCF group, although the difference was not statistically significant (P> 0.05) (Figure 2). In addition, the median PFS was 7.00 (95% CI: 2.42-11.58) months in the m-DCF group and 8.00 (95% CI: 3.97-12.03) months in the EOX group (Figure 3); however, there was no significant difference between the two groups (P> 0.05). Finally, 80.95% of patients from the m-DCF group and 73.68% of patients from the EOX group died. 

*Chemotherapy*

In this study, four (21.05%) patients from the EOX group and five (23.80%) patients from the m-DCF group had a reduction in at least one dose of chemotherapy. Also, six (31.58%) patients from the EOX group and five (23.80%) patients from the m-DCF group experienced delays in at least one treatment cycle due to toxicity induced by chemotherapy; however, the two groups showed no significant difference (P> 0.05).

*Toxicity (secondary endpoint)*

The most common side effect (grade III and IV) in the EOX group was reduction of white blood cell (WBC), neutrophil, and platelet counts (10.53% each), while in the m-DCF group, the most adverse event was reduced neutrophil count (28.57%) (Table 3). Although there was no significant difference in terms of the side effects between the two groups, deep vein thrombosis (DVT) in the EOX group and neutropenic fever in the m-DCF group led to longer hospitals stays. All side effects were well treated with proper management. Overall, 10 (47.61%) patients from the m-DCF group and four (21.05%) patients from the EOX group received supportive treatment during chemotherapy, although there was no significant difference between the groups (P= 0.08) (Table 4).

**Table 1:** Demographic and Baseline Characteristics of Study Participants

Variables	m-DCF (21)	EOX (19)	P
Mean age	54.10±15.21	62.63±14.70	0.08
Gender (M, F)	11, 10	14, 5	0.20
Gastrectomy history	14	9	0.34
Locally advanced	11	10	1.00
metastasis	10	9	1.00
Metastasis location			
lung	1	0	1.00
Liver	2	7	0.06
Pancreas	1	0	1.00
Distant lymph nodes	1	1	1.00
Multiple organs	1	0	1.00
Lauren classification			0.49
Intestinal	3	3	
Diffuse	6	7	
unknown	12	9	

**Table 2:** Comparison of Efficacy in m-DCF and EOX Groups in Patients with Advanced Gastric Cancer

	m-DCF	EOX	P
Median overall survival, months (95 % CI)	14.00 (CI 95%: 11.82-16.18)	15.00 (CI 95%: 9.56- 20.43)	0.65
2-year survival rate, %	27	29	
Median progression-free survival, months (95 % CI)	7.00 (2.42-11.58)	8.00 (3.97-12.03)	0.32
At least one dose reduction	5	4	1.00
At least one cycle delay	5	6	0.73

**Table 3:** Comparison of Grade 3 or 4 Toxicity in m-DCF and EOX groups in Advanced Gastric Cancer Patients

Variable	m-DCF	EOX	P
Anemia	2	1	1.00
White blood cell decreased	2	2	1.00
Neutrophil count decreased	6	2	0.23
Febrile nutropenia	1	0	1.00
Platelet count decreased	1	2	0.60
Weight loss	2	0	0.49
Dyspnea	2	0	0.49
Abdominal pain	1	0	1.00
Spinal fracture	0	1	0.48
Deep vein thrombosis	0	1	0.48
Diarrhea	0	1	0.48
Vomiting	2	0	0.49

**Table 4:** Comparison of the Need for Supportive Treatment in Advanced Gastric Cancer Patients in m-DCF and EOX Groups

	m-DCF	EOX	P
erythropoietin	9	4	0.17
G-CSF	6	2	0.23
Blood transfusion	2	1	0.57

## Discussion

Despite many challenges in the treatment and management of gastric cancer, three-drug regimens, consisting of mDCF and EOX, seem to significantly improve the survival of patients. However, there are still some controversies regarding the efficacy, safety, tolerability, and cost-effectiveness of these regimens. Recently, several trials have indicated that m-DCF and EOX regimens are better options for the standard treatment of patients with stage IV gastric cancer, compared with other regimens. Therefore, in this study, we investigated the efficacy and safety of m-DCF versus EOX regimen in advanced gastric cancer (stage IV) patients with the median follow-up of 24-months.

The results of the present study showed that the median survival was 14.00 (95% CI: 11.82-16.18) months in the m-DCF group and 15.00 (95% CI: 9.56-20.43) months in the EOX group. Although survival was longer in the EOX group, there was no significant difference between the two groups. Also, the mean PFS was 7.00 (CI 95%:2.42-11.58) months in the m-DCF group and 8.00 (CI 95%:3.97-12.03) months in the EOX group; however, there was no significant difference between the groups. 

In this regard, Ochenduszko et al., (2015) in a similar trial compared m-DCF and EOX regimens in gastric cancer patients. Their findings showed that the median survival was 11.9 months in the m-DCF group and 9.5 months in the EOX group. Although survival was longer in the m-DCF regimen, there was no significant difference between the groups, which is similar to our study. In addition, Ochenduszko et al. showed that PFS was 6.8 months in the m-DCF group and 6.4 months in the EOX group, which is consistent with our study. 

Moreover, Inal et al., (2012) in a retrospective study during 2007-2011, determined the effectiveness and tolerability of m-DCF regimen. Their findings showed that the median PFS was 6.5 months in the m-DCF group, while the median OS was 8.6 months. Kang et al., (2011) in a similar clinical trial study, also showed that the median survival of gastric cancer patients under treatment with m-DCF was 14.4 months, and PFS was 7.6 months. Additionally, Shah et al., (2015) in agreement with our study, reported a median OS of 18.8 months and a median PFS of 9.7 months in m-DCF-treated patients.

In a clinical trial conducted by Cunningham et al., (2008) in England on various regimens, including epirubicin, cisplatin, and fluorouracil (ECF), epirubicin, cisplatin, and capecitabine (ECX), epirubicin, oxaliplatin, and fluorouracil (EOF), and EOX, the highest survival rates were reported in the EOX group (median OS: 11.2 months). The results of this trial showed that the EOX regimen significantly improved patient survival, compared with the ECF regimen. Other advantages of EOX regimen, compared with the ECF regimen, include better tolerability, fewer thromboembolic events, and lack of need for a central catheter insertion (unlike DCF regimen) (Cunningham et al., 2008). The results of this trial indicated that there was no significant difference in terms of the side effects between the two treatment regimens, as confirmed in the study by (Ochenduszko et al., 2015).

In our study, the most common side effect in the EOX group was reduction of WBC, neutrophil, and platelet count (10.53%). In a clinical trial by Xiang et al. on advanced gastric cancer patients in China, the most common toxicity was grade III or IV neutropenia (22.9%) in the EOX regimen, which is almost similar to the present study (Xiang et al., 2010). In another study, Cunningham et al. showed that the most common toxicity was grade III/IV neutropenia in the EOX group (27.6%) (Cunningham et al., 2008). In the study by Ochenduszko et al., the frequency of grade III/IV neutropenia in patients treated with the EOX regimen was 72.4% (Ochenduszko et al., 2015).

One of the important findings regarding thromboembolic toxicity in our study is that it only occurred in the EOX group. The study by Ochenduszko et al., (2015) showed that the rate of thromboembolic events in the EOX group was about twice as high as the m-DCF group (13.8% vs. 7.7%), which suggests that the EOX regimen should be avoided as much as possible in patients with a high risk of thromboembolic events in order to avoid these outcomes and their subsequent negative effects on the survival of patients; however, further studies are needed to confirm this relationship (Khorana et al., 2007; Tetzlaff et al., 2008).

In the present study, the most common side effect in the m-DCF group was grade III and IV neutropenia (28.57%). Unlike our study, Ochenduszko et al., (2015) showed that neutropenia was more frequent in the EOX group, compared with the m-DCF group. On the other hand, in our study, most cases of neutropenia occurred in the m-DCF group. Although the differences were not significant in these studies, the increased incidence of neutropenic events in our study might be attributed to the lack of granulocyte colony-stimulating factor (G-CSF) in the early stages of neutropenia and limited G-CSF administration. In the current study, G-CSF was prescribed only in 29% of patients, while in the study by Ochenduszko et al., (2015) G-CSF was prescribed in 55.6% of patients in the m-DCF group.

In another study by Shah et al., the most common side effects (grade III or IV) in the m-DCF group were neutropenia (56%), leukopenia (44%), and thromboembolism (20%) (Shah et al., 2015). Inal et al., (2012) also reviewed the effectiveness and tolerability of m-DCF regimen. They found that the most common grade III and IV complications in the m-DCF group were neutropenia (13.6%), nausea (13.6%), anemia (4.5%), and vomiting (4.5%), which is similar to our study in some aspects.

*Limitations*

This study had several limitations, the most important of which is the small sample size. Other limitations of this study include the single-center design and lack of patient assessment regarding HER2 receptor status, nutritional status, and body mass index as prognostic factors. Considering these limitations, generalizability of our findings should be done with caution. We recommend further multi-center clinical trials with a larger sample size, longer follow–up, and quality of life assessment.

The results of this study revealed that there is no significant difference between the m-DCF and EOX regimens regarding survival, PFS, and side effects. However, considering the need for hospitalization due to continuous infusion of 5-fluorouracil and lack of admission in the EOX group, this regimen seems to be more acceptable in patients who are unwilling to be hospitalized.
